# Safety and efficacy of renal sympathetic denervation: a 9-year long-term follow-up of 24-hour ambulatory blood pressure measurements

**DOI:** 10.3389/fcvm.2023.1210801

**Published:** 2023-06-19

**Authors:** Alexander Vogt, Jochen Dutzmann, Michael Nußbaum, Daniel Hoyer, Jörn Tongers, Axel Schlitt, Daniel Sedding, Alexander Plehn

**Affiliations:** ^1^ Department of Internal Medicine III, University Hospital Halle (Saale), Halle (Saale), Germany; ^2^ Department of Cardiology and Diabetes, Paracelsus-Harz-Clinic Bad Suderode, Quedlinburg, Germany; ^3^ Praxisklinik Salzatal, Salzatal, Germany

**Keywords:** renal denervation (RDN), uncontrolled hypertension, renal function, ambulatory blood pressure, long-term effect

## Abstract

**Background:**

Renal sympathetic denervation (RDN) has been shown to lower arterial blood pressure both in the presence and in the absence of antihypertensive medication in an observation period of up to 3 years. However, long-term results beyond 3 years are scarcely reported.

**Methods:**

We performed a long-term follow-up on patients who were previously enrolled in a local renal denervation registry and who underwent radiofrequency RDN with the Symplicity Flex® renal denervation system between 2011 and 2014. The patients were assessed to evaluate their renal function by performing 24-hour ambulatory blood pressure measurement (ABPM), recording their medical history, and conducting laboratory tests.

**Results:**

Ambulatory blood pressure readings for 24 h were available for 72 patients at long-term follow-up (FU) [9.3 years (IQR: 8.5–10.1)]. We found a significant reduction of ABP from 150.1/86.1 ± 16.9/12.0 mmHg at baseline to 138.3/77.1 ± 16.5/11.1 mmHg at long-term FU (*P *< 0.001 for both systolic and diastolic ABP). The number of antihypertensive medications used by the patients significantly decreased from 5.4 ± 1.5 at baseline to 4.8 ± 1.6 at long-term FU (*P* < 0.01). Renal function showed a significant but expected age-associated decrease in the eGFR from 87.8 (IQR: 81.0–100.0) to 72.5 (IQR: 55.8–86.8) ml/min/1.73 m^2^ (*P *< 0.01) in patients with an initial eGFR > 60 ml/min/1.73 m^2^, while a non-significant decrease was observed in patients with an initial eGFR < 60 ml/min/1.73 m^2^ at long-term FU [56.0 (IQR: 40.9–58.4) vs. 39.0 (IQR: 13.5–56.3) ml/min/1.73 m^2^].

**Conclusions:**

RDN was accompanied by a long-lasting reduction in blood pressure with a concomitant reduction in antihypertensive medication. No negative effects could be detected, especially with regard to renal function.

## Introduction

High blood pressure remains one of the leading cardiovascular risk factors and the leading cause of premature death, affecting more than 30% of the adult population worldwide (1). Various guidelines recommend the treatment of patients diagnosed with hypertension (2–4) since the relationship between elevated blood pressure and premature cardiovascular events and death is well established (5).

In patients in whom secondary causes for hypertension, which need specific treatments, can be excluded, the guidelines recommend lifestyle changes and, depending on additional risk factors and hypertension severity, antihypertensive medication (2–4). Nevertheless, despite numerous treatment modalities, a relevant proportion of medically treated patients do not achieve the recommended blood pressure reduction targets (6). Patients with treatment-resistant hypertension have a substantially higher risk for adverse cardiovascular events such as myocardial infarction, heart failure, stroke, chronic kidney disease, or death when compared with patients whose treatment targets can be achieved (7).

In recent randomized sham-controlled trials, renal denervation (RDN) has been shown to effectively reduce blood pressure in the absence or presence of antihypertensive medication over a period of up to 6 months (8–12). Moreover, the long-term results of the large randomized sham-controlled SYMPLICITY HTN-3 trial (13) and the SPYRAL HTN-ON MED trial (14) demonstrated a relevant antihypertensive effect over a 3-year period after RDN. However, long-term data beyond the 3-year period are scarcely available.

The aim of this analysis is to evaluate the long-term efficacy and safety of radiofrequency RDN in a cohort of patients with treatment-resistant hypertension, with a particular focus on the course of blood pressure in patients who initially did not respond to RDN treatment.

## Methods

### Study population

After obtaining approval from the local ethics committee, we identified and contacted 245 patients who were previously enrolled in a local renal denervation (RDN) registry and who underwent radiofrequency RDN with the Symplicity Flex® renal denervation system at the University Hospital Halle (Saale), Germany, between 2011 and 2014 (www.drks.de; identifier: DRKS00004173). All patients underwent baseline evaluation through 24 h ambulatory blood pressure (ABP) measurement and laboratory tests prior to renal denervation. A total of 108 patients were available for long-term follow-up (FU). For 72 of these patients, complete 24 h ABP measurements were available. Patients who declined an on-site visit and ABP measurement (ABPM) were asked to take a telephonic interview and undergo ABPM and laboratory tests through their primary care physician. This study was approved by the local ethics committee.

All patients provided informed consent to this study.

### Renal denervation procedure

Technical and procedural details of ablation systems have been described elsewhere. (15). The procedure was performed by a single experienced operator (AP), who used the Symplicity Flex® renal denervation system by following the instructions for use and recommendations of the device manufacturer. Accessory renal arteries were treated if the length and diameter were found suitable.

### Ambulatory blood pressure measurements and clinical evaluation at long-term FU

For on-site FU of patients, 24 h ABP readings were done using standardized techniques and validated equipment (Mobil-o-Graph®, AMEDTEC GmbH, Germany) according to guideline recommendations (3, 16). The equipment was applied on-site and patients were instructed to leave the system in place for the purpose of measuring a full day–night cycle. ABP and heart rate were measured in intervals of 20 min from 6 a.m. to 10 p.m. and in intervals of 30 min from 10 p.m. to 6 a.m. Electrocardiogram was recorded prior to the use of the ABP equipment.

Blood samples were drawn to determine the electrolyte, creatinine, urea, and HbA1c levels.

Spot urine tests for determining the albumin and creatinine levels were completed during FU of the original registry up to 12 months of FU.

Patients who were not willing or able to complete an on-site visit at long-term FU were asked to have the relevant laboratory tests, electrocardiogram, and ABPMs performed by their primary care physician. Antihypertensive medication was recorded and divided into nine classes [renin–angiotensin–aldosterone system-inhibitors (ACE inhibitors, angiotensin receptor antagonists, and renin inhibitors), calcium channel blockers, beta blockers, diuretics, mineralocorticoid-receptor antagonists, alpha-adrenergic blockers, centrally acting sympatholytic drugs, direct-acting vasodilators, and other medications].

### Endpoints

The primary efficacy endpoint was the change in mean systolic ABP at long-term FU. The safety endpoints were the change in renal function [the estimated glomerular filtration rate (eGFR) was calculated by using the CKD-EPI equation] and new-onset renal artery stenosis.

Early response to RDN treatment was defined as a reduction in mean systolic ABP ≥ 5 mmHg at a 3-month FU.

### Statistical analysis

Continuous symmetrically distributed variables are presented as means ± standard deviation and confidence intervals. Between-group differences were compared using the *t*-test. The median and the 25% and 75% quartiles were calculated to describe skewed variables. The between-group differences of these variables were compared using the Mann–Whitney test. Normal distribution was tested using the Kolmogorov‒Smirnov test. Between-group differences in categorical variables were compared using the *χ*^2^ test. Categorical variables between baseline and long-term FU were compared by using McNemar's test. Urine albumin levels between baseline and FU were compared by using the signed-rank test.

To evaluate the changes in systolic and diastolic ABP, the glomerular filtration rate during FU and linear mixed-effects models were used to account for repeated observations in the same patients over time.

To identify independent correlates of the early response to RDN treatment (as defined above), the following baseline characteristics were assessed in a one-step multivariate binary logistic regression analysis: age, male gender, BMI, number of ablations, systolic ABP, diastolic ABP, mean heart rate, coronary artery disease, atrial fibrillation, current smoking, diabetes mellitus, number of prescribed antihypertensive medications, eGFR, BNP > ULN, and HbA1c.

In case of missing observations in any of the recorded variables, all other available follow-up observations were included in the calculations. No imputation of missing data was done. All endpoints were analyzed exploratively.

A score of *P* ≤ 0.05 was considered statistically significant. Statistical analyses were performed by using SPSS Version 28 (IBM, Armonk, USA), and GraphPad Prism version 9 (GraphPad Software, San Diego, California, USA).

## Results

### FU and patient characteristics

**Figure 1:**
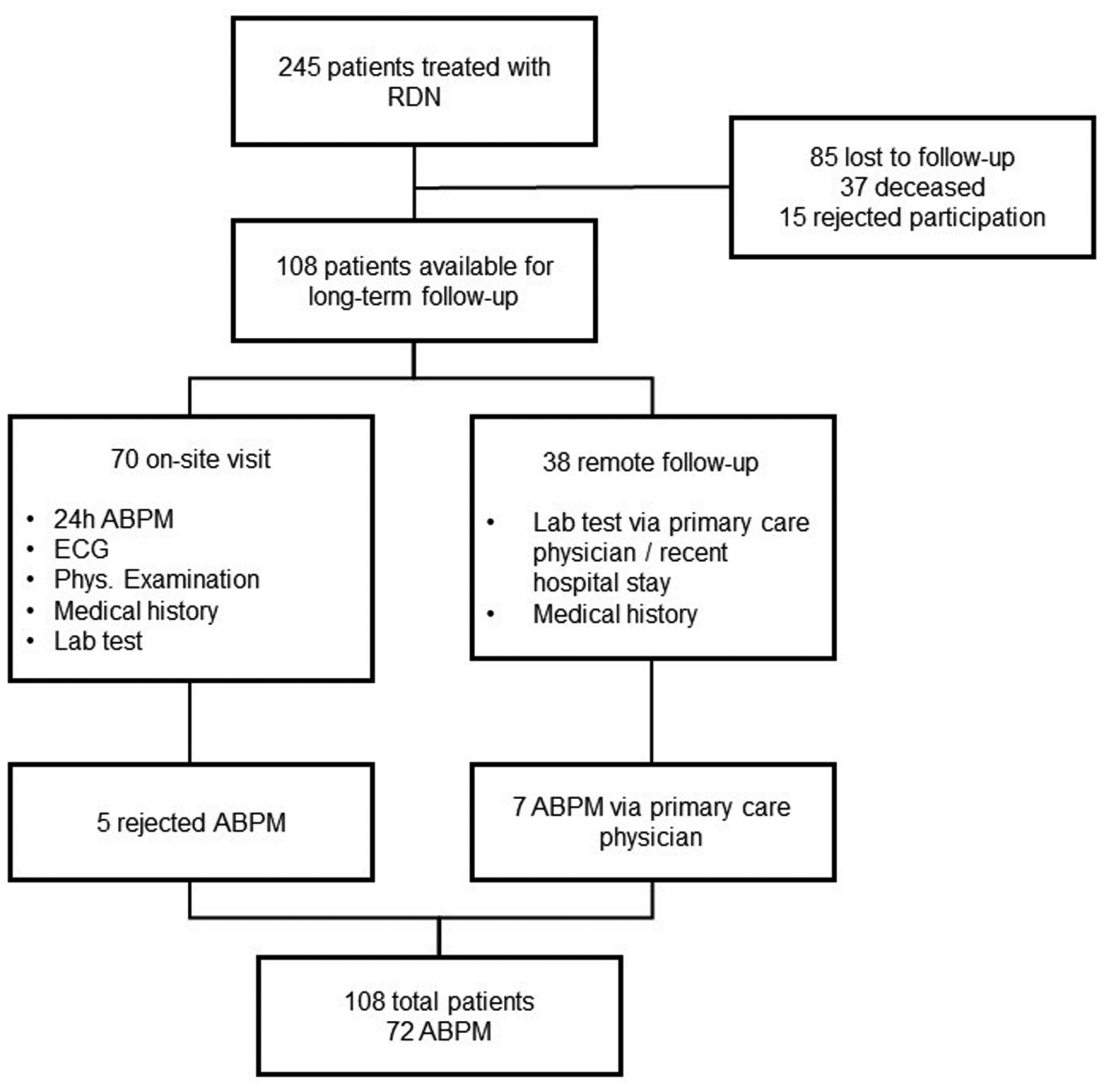
Study overview.

From a total of 245 potential participants, data for 108 patients were available for this analysis. A total of 85 patients were lost to FU, 15 declined participation, and 37 were deceased (Figure 1). Of the 108 patients, 70 opted for on-site visits and 38 for remote FU. ABPM recordings were available for 72 patients. The main reason for patients declining participation or on-site visit was related to long travel distances or immobility. In addition, long-term FUs took place during the COVID-19 pandemic, and therefore, contact restrictions or risk of infection were additional reasons mentioned for declining participation.

At the time when the procedure was carried out, the patients were 63.2 ± 8.8 years old, and 60 of them (55.6%) were male.

The patients were mildly obese with a BMI of 30.9 ± 5.1 kg/m^2^, which increased slightly but significantly to 31.6 ± 5.4 during long-term FU. Atrial fibrillation (paroxysmal, persistent, long-standing persistent, or permanent) was noticed in 11.1% of patients at baseline and 35.2% at long-term FU (*P *< 0.01). The proportion of patients with chronic kidney disease (eGFR < 60 ml/min/1,73 m^2^) increased from 10.2% to 33.3% (*P *< 0.01).

Of the patients, 13.9% had known coronary artery disease. Diabetes mellitus was present in 43.5% of patients. The patients were treated with 12.4 ± 3.1 ablations. The baseline characteristics are given in Table 1.

**Table 1:** Patient characteristics at baseline and long-term follow-up.

Clinical characteristics	Baseline	Long-term FU	*P*-value
Age (years)	63.2 (±8.8)	72.2 (±8.5)	<0.001
Male, *n* (%)	60 (55.6%)	60 (55.6%)	NS
BMI (kg/m^2^)	30.9 (±5.1)	31.6 (±5.4)	0.04
Number of ablations (*n*)	12.4 (±3.1)	–	
Blood pressure measurement			
24 h ABP sys (mmHg)	150.1 (±16.9)	138.3 (±16.5^a^	<0.001
Day	153.4 (±17.4)	140.5 (±15.4)^a^	<0.001
Night	143.3 (±19.8)	134.1 (±20.6)^a^	0.007
24 h ABP dia (mmHg)	86.1 (±12.0)	77.1 (±11.1)^a^	<0.001
Day	89.1 (±12.3)	79.6 (±10.4)^a^	<0.001
Night	79.4 (±12.8)	72.9 (±12.0)^a^	<0.001
Dipping, *n* (%)	36 (33.3%)	18 (25.0%)^a^	NS
24 h heart rate (bpm)	64.2 (±9.9)	66.3 (±12.1)^a^	NS
Medical history			
Coronary artery disease	15 (13.9%)	19 (17.6%)	NS
Atrial fibrillation	12 (11.1%)	38 (35.2%)	<0.001
Currently smoking	41 (38.0%)	42 (38.9%)	NS
Diabetes mellitus	47 (43.5%)	52 (48.1%)	NS
Chronic kidney disease (eGFR < 60 ml/min/1.73 m²)	11 (10.2%)	36 (33.3%)	<0.001
Laboratory			
Plasma creatinine (µmol/L)	77.0 (66.3–89.0)^b^	89.0 (77.0–115.3)^b^	<0.001
Glomerular filtration rate (ml/min/1.73 m^2^)	86.3 (74.0–98.1)^b^	68.0 (51.4–84.7)^b^	<0.001
BNP/NT-pro-BNP^c^	40.0 (25.0–90.5)^b^^,^^c^	186.5 (106.8–65.5)^b^^,^^c^	–
BNP > ULN, *n* (%)	23 (21.3%)	48 (44.4%)	<0.001
HbA1c (mmol/mol)	46.0 (±12.2)	46.0 (±10.2)	NS

ABP, ambulatory blood pressure; BMI, body mass index; bpm, beats per minute; BNP > ULN, brain natriuretic peptide at baseline and NT-proBNP, at long-term follow-up over the upper limit of normal; eGFR, estimated glomerular filtration rate.

^a^ ABPM available for 72 patients at long-term FU.

^b^ Indicates skewed variables.

^c^ Given as BNP (pg/ml) at baseline and NT-pro-BNP (pg/ml) at long-term FU.

The median time to FU was 9.3 (IQR: 8.5–10.1) years.

### Blood pressure

Both mean systolic and mean diastolic blood pressure of the overall cohort significantly decreased from baseline to long-term FU (systolic ABP from 150.1 ± 16.9 mmHg to 138.3 ± 16.5 mmHg, *P *< 0.01, and diastolic ABP from 86.1 ± 12.0 to 77.1 ± 11.1, *P* < 0.01).

**Figure 2:**
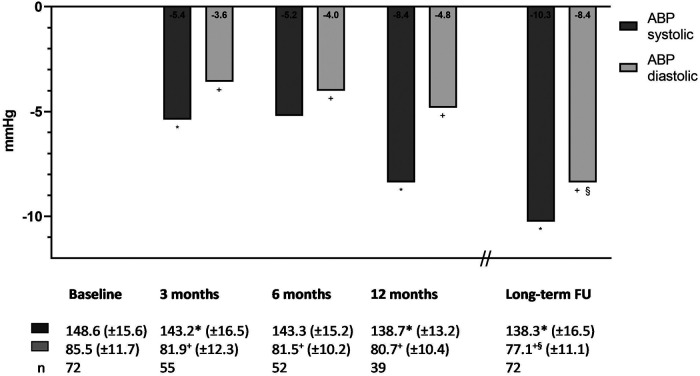
Difference in systolic and diastolic ambulatory blood pressure. *P < 0.05 compared with baseline systolic ABP. ^+^*P* < 0.05 compared with baseline diastolic ABP. ^§^*P* < 0.05 compared with 12-month diastolic ABP.

Changes in systolic and diastolic blood pressure over time of patients whose ABPMs were complete at baseline and FU are shown in Figure 2. At 3 months of FU, mean systolic ABP significantly reduced by −5.4 mmHg to 143.2 ± 16.5 mmHg and diastolic ABP significantly declined by −3.6 mmHg to 81.9 ± 12.3 mmHg (*P *< 0.05 for both). Reduced blood pressure was also evident at all subsequent FU time points (143.3/81.5 ± 15.2/10.2 mmHg at 6 months, 138.7/80.7 ± 13.2/10.4 mmHg at 12 months, and 138.3/77.1 ± 16.5/10.1 mmHg at long-term FU, *P *< 0.05 for all comparisons vs. baseline, and comparison of diastolic ABP at long-term FU vs. 12 months, except for systolic ABP at 6 months compared with baseline).

A total of 38 of 73 patients (52.1%), whose blood pressure readings were available at baseline and at 3-month FU, responded early to RDN treatment (as defined by a reduction in mean systolic blood pressure ≥5 mmHg). When early responders at the 3-month FU were compared with non-responders (*n* = 35, 47.9%), with respect to baseline characteristics, no significant differences were found except for baseline systolic ABP, which was significantly higher in the early responder group (154.8 ± 17.3 vs. 143.8 ± 15.0, *P *< 0.01, Supplementary Table S1), and for the proportion of dippers (defined as a decrease in nighttime systolic blood pressure by ≥10% of daytime systolic blood pressure), which was significantly lower in the group of early responders (23.7% vs. 48.6%, *P *= 0.03).

In the multivariate logistic regression analysis to identify independent correlates of early treatment response (as defined above), it was found that age (Exp(B) 0.85, 95% CI: 0.73–0.99, *P *= 0.03), systolic ABP (Exp(B) 1.09, 95% CI: 1.02–1.17, *P *= 0.01), mean heart rate (Exp(B) 0.92, 95% CI: 0.85–1.00, *P *= 0.04), and dipping (Exp(B) 0.22, 95% CI: 0.05–0.96, *P *= 0.04; Supplementary Table S2) were independent predictors of the early response to RDN treatment.

When applying the model to determine whether there was a clinically meaningful reduction in systolic ABP of 10 mmHg during the long-term FU, it was found that only systolic ABP was an independent predictor of the long-term treatment response (Exp(B) 1.11, 95% CI: 1.02–1.21, *P *= 0.02; Supplementary Table S3).

**Figure 3:**
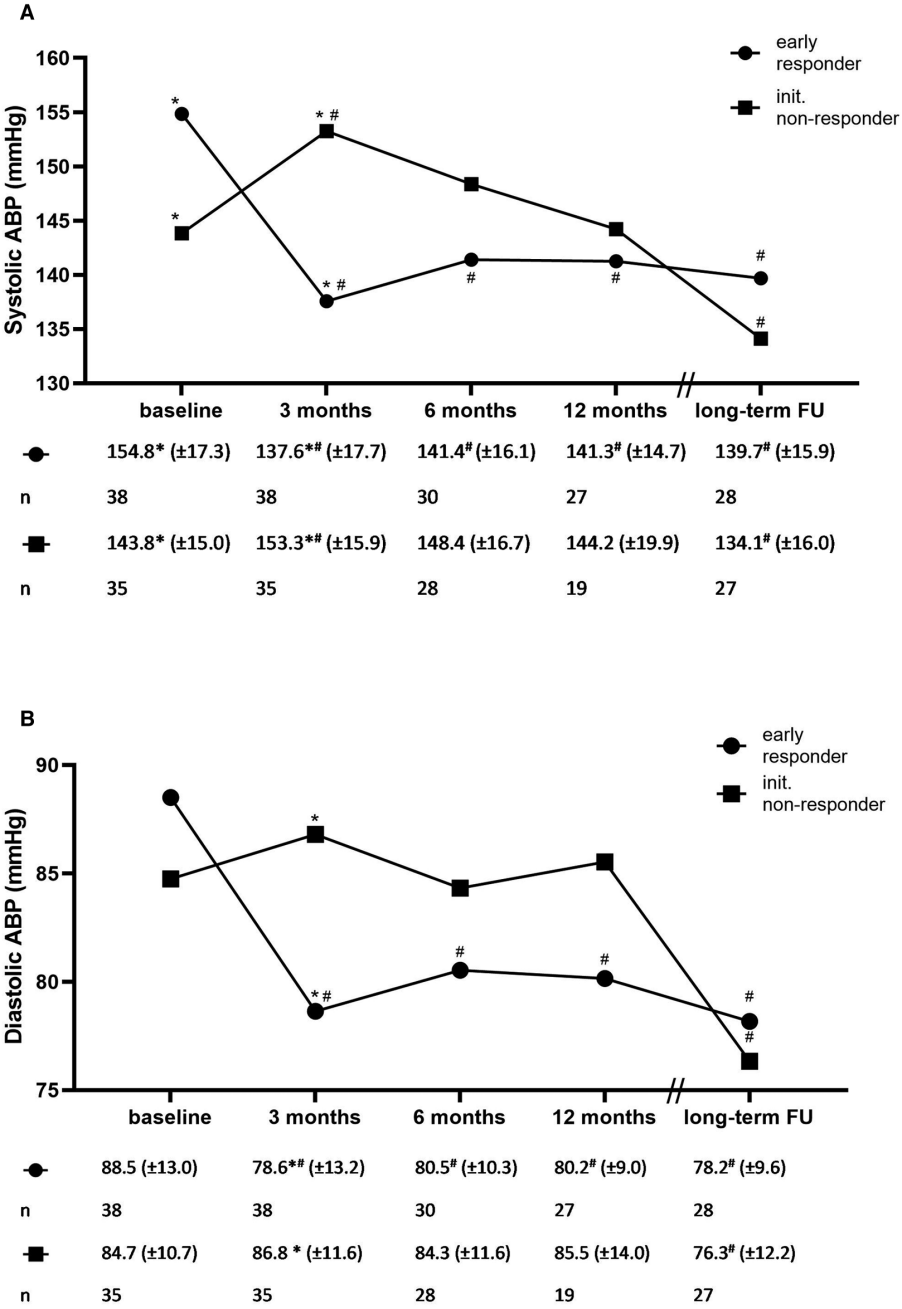
Systolic (**A**) and diastolic (**B**) ambulatory blood pressure and the number of antihypertensive medications for early treatment responders and initial non-responders. ABP: ambulatory blood pressure. *P < 0.05 for between-group comparison. ^#^*P *< 0.05 compared with baseline.

In the comparison of early responders and non-responders, a significantly pronounced reduction in blood pressure was already observed at the 3-month FU in the early responder group, whereas in the non-responder group, a gradual reduction over the follow-up period was observed after an initial increase in mean systolic and diastolic blood pressure. While systolic ABP was significantly higher in the early responder group at baseline, there was no significant difference between the two groups at long-term FU (Figures 3A,B).

### Medication

During the implementation of the RDN procedure, it was found that the patients were taking an average of 5.4 ± 1.5 antihypertensive agents. During long-term FU, the number of antihypertensive agents being consumed decreased by 0.6 to –4.8 ± 1.6 compared with baseline (*P *< 0.01). When comparing patients classified as early responders at 3 months with non-responders, it was found that a slightly but not a significantly higher number of antihypertensive agents was being consumed by non-responders during FU. Between baseline and long-term FU, a non-significant decrease in the number of antihypertensive agents being consumed could be observed in both groups (Supplementary Figure S1).

The medication prescribed at baseline and long-term FU is shown in Table 2. While the proportion of RAAS blockers, calcium antagonists, beta blockers, and diuretics remained constant, the proportion of centrally acting sympatholytic drugs (53.7% vs. 34.4%, *P *< 0.01) and other antihypertensive medication (59.3% vs. 45.8%, *P *= 0.02) decreased significantly. In contrast, the low proportion of MRA at baseline increased significantly at long-term FU (9.3% vs. 29.6%, *P *< 0.01). Patients treated with MRA tended to have lower blood pressure than patients without this therapy; however, this difference was not statistically significant [baseline systolic ABP: 144 (±16) mmHg vs. 151 (±17) mmHg, *P* = 0.29, baseline diastolic ABP: 90 (±10) mmHg vs. 86 (±12) mmHg, *P* = 0.37; long-term FU systolic ABP: 133 (±16) mmHg vs. 141 (±16) mmHg, *P* = 0.09, long-term FU diastolic ABP: 75 (±11) mmHg vs. 78 (±11) mmHg, *P* = 0.36].

**Table 2:** Classes of antihypertensive medications.

	Baseline (*n* = 108)	Long-term FU (*n* = 108)	*P-*value
ACEI/ARB/RI, *n* (%)	105 (97.2)	102 (94.4)	NS
CCB, *n* (%)	78 (72.2)	76 (70.4)	NS
Beta blockers, *n* (%)	92 (85.2)	88 (81.5)	NS
Diuretics, *n* (%)	94 (87.0)	87 (80.6)	NS
MRA, *n* (%)	10 (9.3)	32 (29.6)	<0.01
Alpha-adrenergic blockers, *n* (%)	55 (50.9)	41 (38.0)	NS
Centrally acting sympatholytic drugs, *n* (%)	58 (53.7)	37 (34.4)	<0.01
Direct-acting vasodilators, *n* (%)	15 (13.9)	20 (18.5)	NS
Others, *n* (%)	64 (59.3)	49 (45.8)	0.02
No. of antihypertensive medications	5.4 (±1.5)	4.8 (±1.6)	<0.01

ACEI, angiotensin-converting enzyme inhibitor; ARB, angiotensin receptor blocker; CCB, calcium channel blocker; MRA, mineralocorticoid-receptor antagonist; RI, renin inhibitor.

### Renal function

**Figure 4:**
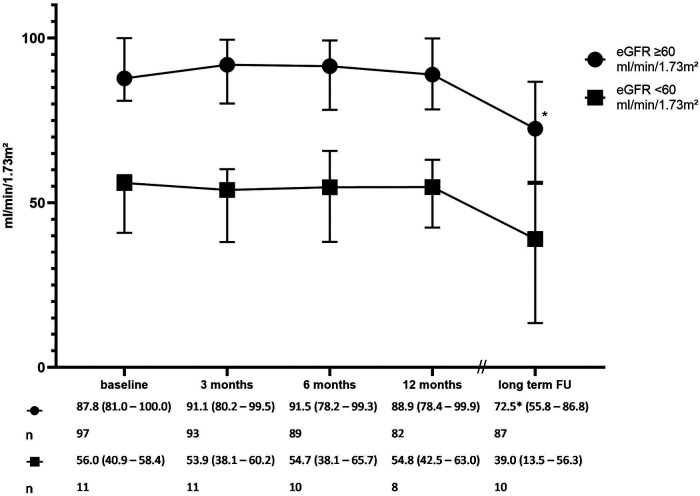
Renal function over time (the glomerular filtration rate estimated by CKD-EPI). The error bars represent the interquartile range. *P < 0.01 compared with baseline. *P* for interaction 0.29.

The renal function of the patients was determined by measuring their plasma creatinine levels. In addition, urine albumin values were available for the first 12 months of FU. At baseline, most patients (89.8%) had a normal renal function (estimated GFR ≥ 60 ml/min/1.73 m^2^) as calculated through the CKD-EPI formula using plasma creatinine with a median eGFR of 87.8 (IQR: 81.0–100.0) ml/min/1.73 m^2^, which remained stable over the FU period up to 12 months. Similarly, the estimated GFR in patients with a baseline eGFR < 60 ml/min/1.73 m^2^ and a median eGFR of 56.0 (IQR: 40.9–58.4) ml/min/1.73 m^2^ was stable up to 12 months after RDN. A decrease in the eGFR was observed at the long-term FU in both groups [72.5 (IQR: 55.8–86.6) ml/min/1.73 m^2^, *P *< 0.01] compared with that at baseline in patients with normal renal function during RDN. There was a non-significant reduction in the eGFR to 39.0 (IQR: 13.5–56.3) ml/min/1.73 m^2^ in the group of patients with impaired renal function during RDN; Figure 4).

Urinary albumin values were available at follow-up time points up to 12 months. In the group of patients with an eGFR ≥ 60 ml/min/1.73 m^2^ during RDN, there was a slight but significant decrease from 8.4 (IQR: 3.2–25.0) mg/L at baseline to 4.8 (IQR: 3.0–13.7) mg/L at 12 months (*P *< 0.01 for comparison with baseline). Urinary albumin levels of patients with an eGFR < 60 ml/min/1.73 m^2^ at the time of ablation increased during the FU [baseline 5.7 (IQR: 4.4–87.7) mg/L and 12 months 13.5 (IQR: 2.9–26.2) mg/L]. However, the changes observed in patients with impaired renal function were not significant (Supplementary Table S4).

### Adverse events

No serious or life-threatening periinterventional complications were observed. A clinically significant bleeding of the arterial access occurred in three patients, which could be treated conservatively.

Of the 245 patients initially enrolled in the registry, 37 died during follow-up. Of these, two died because of heart failure, two because of intracranial hemorrhage, one because of ischemic stroke, six because of malignancies, one because of COVID-19-ARDS, and one because of a traffic accident, according to primary care physicians and family members. The cause of death could not be established in 24 patients. One patient later received additional RDN treatment and one patient was later treated with baroreceptor stimulation. Five patients suffered from non-fatal acute myocardial infarction and 14 patients suffered from non-fatal stroke. Symptomatic heart failure was diagnosed in 39 patients. Postinterventional renal artery stenosis could not be detected in any patient and therefore was not reported.

## Discussion

The results of this analysis are in line with those of recent randomized sham-controlled trials using radiofrequency renal denervation, which demonstrated a decrease in systolic ABP of 7.0 mmHg with concomitant antihypertensive medication at 6 months (10) and of 3.9 mmHg without concomitant antihypertensive medication at 3-month (9) FU, respectively. Such effects have also been shown for ultrasound renal denervation (11, 12). Several register-based studies (17–20), some of them very large, as well as the recently published long-term results of the SYMPLICITY HTN-3 trial (13), were able to show such effects to be even more pronounced over a period of 3 years. A long-term FU of smaller cohorts showed comparable results (21). The results of the present study can complement these findings. We found a significant blood pressure–lowering effect from 150.1/86.1 ± 16.9/12.0 mmHg at baseline to 138.3/77.1 ± 16.5/11.1 mmHg at long-term FU. Moreover, in our cohort, the reduction in blood pressure levels did not stop after 12 months, with blood pressure continuing to decrease significantly and being clinically relevant to the long-term FU of 9.3 years.

Interestingly, patients initially classified as non-responders at 3 months showed a decrease in their systolic and diastolic blood pressure levels over a period of time after an initial increase, whereas blood pressure reduction in the early responder group was swift and sustained. The reduction in blood pressure at the long-term FU was even more pronounced in the group of initial non-responders than in the early responder group. However, the difference between these groups was no longer significant. Large randomized trials and registries, most notably, the SYMPLICITCY HTN-3 (13) and the Global SYMPLICITY Registry (17), have also demonstrated a gradual increase in the antihypertensive effect of RDN over a long-term period. However, data for the FU period between 12 months and long-term FU are missing in our study due to methodological reasons, whereas the abovementioned studies cover this period up to 3 years after RDN and can show a positive effect of this treatment on the incidence of cardiovascular events even now (22).

Combining the results presented here with the results of the abovementioned studies throws up the following question: At what postinterventional time point can a positive treatment effect of RDN be assumed? The physiological mechanisms of a (late) response to RDN and the role that reinnervation and remodeling of sympathetic and parasympathetic nerve fibers play in this context are complex, and therefore, have not yet been fully understood (23, 24). From a clinical perspective, it seems reasonable not to judge the success of treatment before 1-year post-intervention.

As in many other reports (17, 19, 20, 25, 26), in the presented cohort, 24-h baseline systolic blood pressure was an independent predictor of both responses to RDN therapy at 3-month FU and at long-term FU in multivariate binary logistic regression analysis.

However, it must be noted that, to date, there has been no uniform definition of (early) treatment response to RDN. Therefore, we decided to use a dichotomous definition of early treatment response for a clinically relevant reduction in systolic ABP by ≥5 mmHg at 3-month FU from baseline systolic ABP as in previous studies (27).

The number of medications taken, 5.4 ± 1.5 at baseline, was higher than the number reported in other studies (17, 20), but, as in these studies, it remained without any significant change over the period of 12 months in the overall cohort. In contrast, over the entire long-term FU period, a clinically relevant decrease can be seen in the overall cohort (5.4 ± 1.5 vs. 4.8 ± 1.6, *P < *0.01). In recent years, MRAs have become increasingly important in the drug therapy of resistant hypertension and have received higher grades of recommendation in the corresponding guidelines (3, 28, 29). In addition, symptomatic heart failure was diagnosed in a relevant number of patients at the long-term FU and the proportion of patients with NT-pro-BNP levels above ULN significantly increased to 44.4%. Consequently, in the composition of antihypertensive therapy of the cohort reported here, there is an increase in the proportion of MRAs, whereas other drug classes such as alpha blockers and central drugs lost importance. Since specific heart failure medications (e.g., sodium-glucose cotransporter-2 inhibitors) were not recorded in detail, in part, this may also have led to a decrease in the number of antihypertensive drugs taken by patients.

In the cohort presented here, no adverse effects on renal function were found as a safety aspect in patients both without and with renal insufficiency (eGFR < 60 ml/min/1.7 m^2^). This finding is consistent with that of other reports demonstrating this over a FU period of 12 (30), 24 (31), and 36 (17) months. At the long-term FU, a decline in renal function was found in both groups, as expected, in the range of 0.9–5.8 ml/min/1.73 m^2^ annually reported for hypertensive patients (32, 33). Although data are available only up to the 12-month FU, data on the present urinary albumin levels also did not show any negative effects on tubular function in the group of patients with either preserved or reduced renal function. In patients with preserved renal function, there was a slight but statistically significant decrease in urine albumin levels [8.4 (IQR: 3.2–25.5) vs. 4.8 (IQR: 3.0–13.7) mg/L].

## Limitations

As is common in registry studies, especially over a long period of time, FU results are available only for a limited proportion of the patients originally included (the number of patients for whom information on the variables given in Table 1 was available at baseline and FU is listed in Supplementary Table S5). The willingness to participate in the long-term FU may have depended on a positive treatment effect, and therefore, an element of bias could have crept into the results in favor of RDN treatment. Particularly with regard to adverse events, missing data may have led to an underestimation of actual event rates. In addition, we did not require imaging to detect new-onset renal artery stenosis, and therefore, the actual number may have been higher. Nevertheless, in our patient cohort, there was no finding that indicated an increased incidence of clinically relevant renal artery stenosis after RDN.

Compared with the registry of randomized controlled trials, this registry includes a more heterogeneous population. However, it represents a real-world collective. The lack of a control group is also a limitation of this study. Adherence to antihypertensive drug therapy was not assessed. However, recent data from controlled trials indicate that the effect of RDN is independent of the medication taken or of adherence (8, 10, 12, 34).

## Conclusion

In this analysis, a long-lasting and profound ABP reduction was seen with a concomitant reduction in antihypertensive medications. Moreover, the results provide an additional indication that RDN can sustainably and progressively lower blood pressure over a period of more than 3 years, even in patients who do not initially respond to the therapy, underscoring its importance as a complementary treatment for arterial hypertension. Also in the long term, no negative effects of RDN can be detected, especially with regard to renal function. Therefore, RDN can be considered an effective, long-lasting, and safe treatment method for arterial hypertension combined with lifestyle modification and drug therapy.

## Data Availability

The datasets generated and analyzed during the current study are available from the corresponding author upon reasonable request. Requests to access the datasets should be directed to alexander.vogt@uk-halle.de.
