# Supplementary material for: Safety and efficacy of renal sympathetic denervation: a 9-year long-term follow-up of 24-hour ambulatory blood pressure measurements
Source: Front Cardiovasc Med. 2023 Jun 19;10:1210801. doi: 10.3389/fcvm.2023.1210801 (PMC10315532; doi:10.3389/fcvm.2023.1210801)
Supplement: Supplementary file 1 [file Table1.docx]

**Supp. Table 1**

| **Clinical baseline characteristics of early responders and initial non-responders** | | | | |
| --- | --- | --- | --- | --- |
|  | | *Early Responder (n=38)* | *Init. Non-responder (n=35)* | *P* |
| Age (years) | | 61.4 (±9.6) | 63.7 (±8.0) | NS |
| Male, n (%) | | 21 (55.3%) | 20 (57.1%) |  |
| BMI (kg/m²) | | 30.4 (±4.7) | 31.1 (±5.3) | NS |
| Number of ablations (n) | | 11.8 (±3.3) | 12.1 (±2.7) | NS |
| *Blood pressure meassurement ** | |  |  |  |
| 24h ABP sys (mmHg)* | | 154.8 (±17.3) | 143.8 (±15.0) | <0.01 |
|  | Day | 158.1 (±17.9) | 147.17 (±15.5) | <0.01 |
|  | Night | 148.1 (±19.4) | 137.2 (±19.0) | 0.02 |
| 24h ABP dia (mmHg)* | | 88.5 (±13.0) | 84.8 (±10.7) | NS |
|  | Day | 91.4 (±13.3) | 88.0 (±11.2) | NS |
|  | Night | 82.3 (±12.6) | 77.6 (±12.1) | NS |
| Dipping, n (%)* |  | 9 (23.7%) | 17 (48.6%) | 0.03 |
| 24h heart rate (bpm)* | | 64.0 (±10.5) | 65.5 (±10.6) | NS |
| *Medical history* | |  |  |  |
| Coronary artery disease | | 5 (13.2%) | 4 (11.4%) | NS |
| Atrial fibrillation | | 3 (7.9%) | 5 (14.3%) | NS |
| Current Smoking | | 13 (34.2%) | 13 (37.1%) | NS |
| Diabetes mellitus | | 14 (36.8%) | 14 (40.0%) | NS |
| Chronic kidney disease (eGFR <60ml/min/1.73m²) | | 4 (10.5%) | 2 (5.7%) | NS |
| # of antihypertensive medications | | 5.3 (±1.6) | 5.6 (±1.6) | NS |
| *Laboratory* | |  |  |  |
| Plasma Creatinin (µmol/l) | | 77.0 (66.8 – 91.5) | 76.0 (65.0 – 87.0) | NS |
| Albumin urine (mg/l) | | 7.0 (3.1 – 29.3) | 6.8 (3.5 – 21.5) |  |
| Glomerular filtration rate (ml/min/1.73m²) | | 85.3 (75.1 – 96.7) | 84.8 (73.9 – 101.4) | NS |
| BNP (pg/ml) | | 37.0 (19.8 – 95.3) | 40.5 (29.0 – 84.8) | NS |
| BNP > ULN (n, %) | | 9 (23.7%) | 7 (20.0%) | NS |
| HbA1c (mmol/mol) | | 43.8 (±11.1) | 43.8 (±9.7) | NS |

**Supp. Table 2**

| **Univariate binary logistic regression analysis for treatment response (reduction of SBP ≥5mmHg) at 3 months follow up** | | | |
| --- | --- | --- | --- |
| ***Clinical characteristics*** | ***Exp(B)*** | ***95% CI for Exp(B)*** | ***P*** |
| Age | 0.97 | 0.92 – 1.02 | 0.28 |
| Male | 0.93 | 0.37 – 2.34 | 0.87 |
| BMI | 0.97 | 0.89 – 1.07 | 0.56 |
| Number of ablations | 0.97 | 0.83 – 1.13 | 0.70 |
| ***Blood pressure meassurement*** |  |  |  |
| 24h ABP sys | 1.05 | 1.01 – 1.08 | <0.01 |
| 24h ABP dia | 1.03 | 0.99 – 1.07 | 0.19 |
| Dipper | 0.30 | 0.11 – 0.84 | 0.02 |
| 24h heart rate | 0.99 | 0.94 – 1.03 | 0.53 |
| ***Medical history*** |  |  |  |
| Coronary artery disease | 1.17 | 0.29 – 4.78 | 0.82 |
| Atrial fibrillation | 0.51 | 0.11 – 2.33 | 0.39 |
| Current Smoking | 0.88 | 0.34 – 2.30 | 0.79 |
| Diabetes mellitus | 0.88 | 0.34 – 2.25 | 0.78 |
| # of antihypertensive medications | 0.91 | 0.68 – 1.22 | 0.54 |
| ***Laboratory*** |  |  |  |
| Glomerular filtration rate (ml/min/1.73m²) | 1.00 | 0.97 – 1.02 | 0.72 |
| BNP | 1.00 | 0.99 – 1.01 | 0.83 |
| HbA1c | 1.00 | 0.96 – 1.05 | 1.00 |
| **Multivariate binary logistic regression analysis for treatment response (reduction of SBP ≥5mmHg) at 3 months follow up** | | | |
| Age | 0.85 | 0.73 – 0.99 | 0.03 |
| 24h ABP sys | 1.09 | 1.02 – 1.17 | 0.01 |
| 24h heart rate | 0.92 | 0.85 – 1.00 | 0.04 |
| Dipping | 0.22 | 0.05 – 0.96 | 0.04 |

**Supp. Table 3**

| **Univariate binary logistic regression analysis for reduction of SBP ≥10mmHg at long term follow up** | | | |
| --- | --- | --- | --- |
| ***Clinical characteristics*** | ***Exp(B)*** | ***95% CI for Exp(B)*** | ***P*** |
| Age | 1.02 | 0.96 – 1.08 | 0.51 |
| Male | 1.88 | 0.73 – 4.89 | 0.19 |
| BMI | 1.04 | 0.91 – 1.11 | 0.93 |
| Number of ablations | 1.18 | 0.97 – 1.44 | 0.09 |
| ***Blood pressure meassurement*** |  |  |  |
| 24h ABP sys | 1.09 | 1.04 – 1.15 | <0.01 |
| 24h ABP dia | 1.06 | 1.01 – 1.12 | 0.03 |
| Dipper | 0.43 | 0.16 – 1.19 | 0.11 |
| 24h heart rate | 1.03 | 0.98 – 1.07 | 0.30 |
| ***Medical history*** |  |  |  |
| Coronary artery disease | 2.19 | 0.52 – 9.25 | 0.29 |
| Atrial fibrillation | 0.39 | 0.07 – 2.29 | 0.30 |
| Current Smoking | 0.93 | 0.37 – 2.36 | 0.87 |
| Diabetes mellitus | 3.11 | 1.15 – 8.39 | 0.03 |
| # of antihypertensive medications | 1.37 | 0.98 – 1.92 | 0.06 |
| ***Laboratory*** |  |  |  |
| Glomerular filtration rate (ml/min/1.73m²) | 1.01 | 0.98 – 1.04 | 0.47 |
| BNP | 1.01 | 1.00 – 1.03 | 0.08 |
| HbA1c | 1.02 | 0.98 – 1.06 | 0.48 |
| **Multivariate binary logistic regression analysis for reduction of SBP ≥10mmHg at long term follow up** | | | |
| 24h ABP sys (mmHg) | 1.11 | 1.02 – 1.21 | 0.02 |

**Supp. Table 4**

| **Urine albumin over time (mg/l)**  (glomerular filtration rate estimated by CKD-EPI)  **P*<0.01 compared to baseline. | | | | |
| --- | --- | --- | --- | --- |
|  | baseline | 3 months | 6 months | 12 months |
| eGFR≥60 ml/min/1.73m² | 8.4 (3.2 – 25.0)  n=88 | 6.0 (3.1 – 17.5)  n=91 | 5.5 (2.9 – 12.5)  n=90 | 4.8* (3.0 – 13.7)  n=80 |
| eGFR<60 ml/min/1.73m² | 5.7 (4.4 – 87.7)  n=9 | 7.7 (3.5 – 175.0)  n=11 | 5.0 (2.4 – 45.0)  n=10 | 13.5 (2.9 – 26.2)  n=8 |

**Supp. Table 5** Number of patients for whom information on the respective variable was available at baseline and follow-up.

| *Clinical characteristics* | | *Baseline* | *Long-term FU* |
| --- | --- | --- | --- |
| Age | | 108 | 108 |
| Male | | 108 | 108 |
| BMI | | 108 | 106 |
| Number of ablations | | 108 | - |
| *Blood pressure meassurement* | |  |  |
| 24h ABP | | 108 | 72 |
| Dipping |  | 108 | 72 |
| 24h heart rate |  | 108 | 72 |
| *Medical history* | |  |  |
| Coronary artery disease | | 108 | 108 |
| Atrial fibrillation | | 108 | 108 |
| Current Smoking | | 108 | 103 |
| Diabetes mellitus | | 108 | 108 |
| Chronic kidney disease (eGFR <60ml/min/1.73m²) | | 108 | 107 |
| *Laboratory* | |  |  |
| Plasma Creatinin | | 108 | 98 |
| Glomerular filtration rate | | 108 | 98 |
| BNP / NT-pro-BNP | | 105 | 74 |
| HbA1c | | 104 | 86 |
