# Supplementary material for: Safety and efficacy of renal sympathetic denervation: a 9-year long-term follow-up of 24-hour ambulatory blood pressure measurements
Source: Front Cardiovasc Med. 2023 Jun 19;10:1210801. doi: 10.3389/fcvm.2023.1210801 (PMC10315532; doi:10.3389/fcvm.2023.1210801)
Supplement: Supplementary file 2 [file Datasheet1.pdf]

Supp. Figure 1. # of antihypertensive medications of early responders and initial non-responders during follow-up

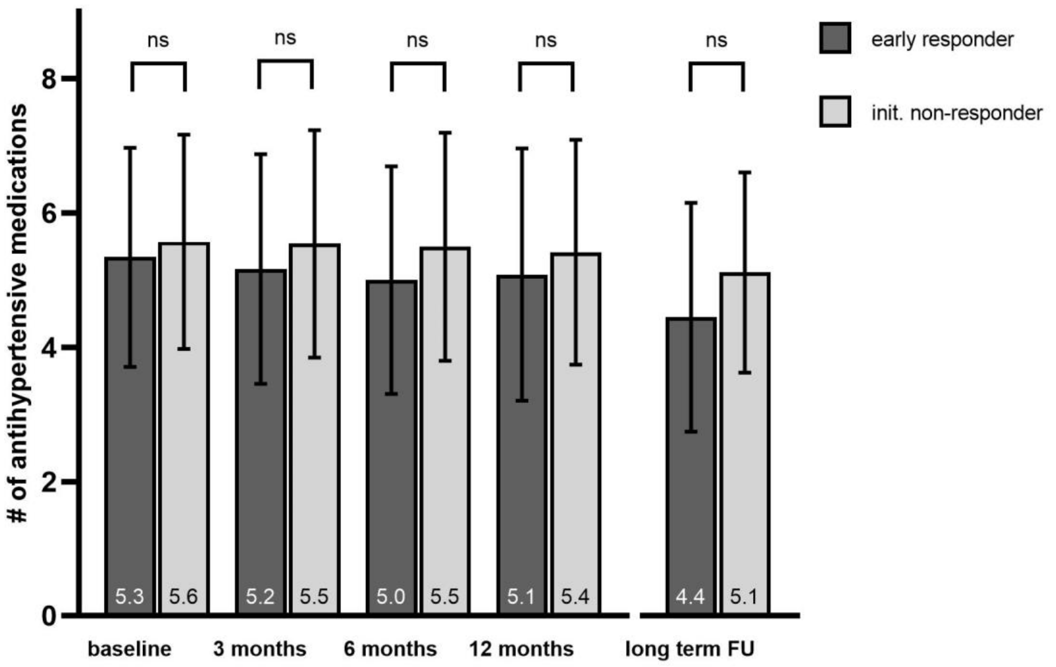

	baseline	3 months	6 months	12 months	long term FU
■ (dark)	5.3 (±1.6)	5.2 (±1.7)	5.0 (±1.7)	5.1 (±1.9)	4.5 (±1.7)
n	38	24	17	12	38
■ (light)	5.6 (±1.6)	5.5 (±1.7)	5.5 (±1.7)	5.4 (±1.7)	5.1 (±1.5)
n	35	24	14	12	35
